# Patient Perspectives on Kidney Stone Surgery: A Content Analysis of Instagram Posts by Patients Versus Surgeons

**DOI:** 10.1016/j.euros.2023.10.009

**Published:** 2023-11-15

**Authors:** Patrick Juliebø-Jones, Esteban Emiliani, Alba Sierra, Francesco Esperto, Eugenio Ventimiglia, Amelia Pietropaolo, Lazaros Tzelves, Christian Beisland, Bhaskar K. Somani

**Affiliations:** a Department of Urology, Haukeland University Hospital, Bergen, Norway; b Department of Clinical Medicine, University of Bergen, Bergen, Norway; c European Association of Urology Young Academic Urologists Urolithiasis Group, Arnhem, The Netherlands; d Department of Urology, Puigvert Foundation, Autonomous University of Barcelona, Barcelona, Spain; e Department of Urology, Hospital Clinic, University of Barcelona, Barcelona, Spain; f Department of Urology, Campus Biomedico University of Rome, Rome, Italy; g Division of Experimental Oncology, Unit of Urology, Urological Research Institute, IRCCS San Raffaele Hospital, Milan, Italy; h Department of Urology, University Hospital Southampton, Southampton, UK; i Second Department of Urology, National and Kapodistrian University of Athens, Sismanogleio General Hospital, Athens, Greece

**Keywords:** Ureteroscopy, Urolithiasis, Social media, Quality of life

## Abstract

**Background:**

Both clinicians and patients use social media to post about health care issues such as kidney stone disease, but their perspectives may differ.

**Objective:**

To evaluate content and themes regarding kidney stone surgery shared by patients and urologists on Instagram.

**Design, setting, and participants:**

A search was performed on Instagram using the term “kidney stone surgery”. The first 100 posts from individuals who were clearly identified as a patient were assessed. We also assessed 100 posts from self-identified urologists.

**Outcome measurements and statistical analysis:**

A previously published system was applied as a framework for categorizing the information collected. Outcomes of interested included pain, recovery, and costs.

**Results and limitations:**

Some 71% of the patients were female and most of their posts (52%) were shared postoperatively. The most common themes covered in patient posts were the need for multiple operative sessions (45%), pain (43%), and recovery (42%). Other themes included activities of daily life (18%), return to work (11%), nervousness (16%), stent issues (31%), stent on a string (5%), diet and prevention (9%), gratitude for health care services (10%), disease recurrence (18%), and costs (10%). Some 94% of the urologists were male and their posts covered the following domains: recovery (11%), stent issues (3%), pain (1%), stent on a string (1%), gratitude for health care services (1%), and recurrence 1%. Among the posts from urologists, 79% included self-promotion and 52% covered new technology. Overall, 10% contained false information. The majority of the posts with surgical images had no clear statement regarding patient consent (97%).

**Conclusions:**

Kidney stone surgery can affect many areas of a patient’s quality of life. Most of the posts shared by patients were negative. Posts shared by urologists do not reflect the same themes. Moreover, there appears to be poor adherence to European Association of Urology recommendations regarding online professional conduct.

**Patient summary:**

Many patients use social media to share their experiences of kidney stone surgery. Posts are largely related to quality-of-life issues and are mostly negative. While urologists also use social media, the content they post on professional accounts is mostly focused on new technology and career promotion.

## Take Home Message

Patients use social media as a vehicle to communicate their experiences regarding kidney stone surgery. Most experiences shared by patients are surrounding quality of life issues and are negative. Urologists posting on kidney stone surgery tend to focus on new technologies and appear to use the platform to promote their careers.

## 1 Introduction

The burden of kidney stone disease (KSD) has increased worldwide and is reflected in an increase in the volume of surgeries being performed [1], [2]. At the same time, the use of social media has grown exponentially. It is therefore not surprising that social media platforms have become a shared space where individuals can learn about their illnesses and treatments, including kidney stone surgery. It has been reported that in the USA, 74% of individuals who use the internet to learn more about a condition use some form of social media to do so [3]. Therefore, the impact of social media on medical decision-making is likely to be considerable [4].

Social media also serves as a vehicle for patient self-expression, as patients use these online platforms to communicate their medical experiences [5]. As a result, these sites can be home to many different communities who support each other through illnesses. Use of social media is not limited to patients, as clinicians also use these platforms. In a recent survey of American Urological Association members, 28% of respondents reported use of social media for professional purposes [6]. Unfortunately, the medical information shared can often be incorrect and lack scientific evidence [7], a topic that has been the focus of most studies investigating kidney stone surgery on social media [8], [9].

Our aim was to evaluate content and themes shared by patients on social media and compare them with those in posts from urologists.

## 2 Patients and methods

A search was performed on Instagram (Facebook Inc., Menlo Park, CA, USA), a social media platform established in 2010 that has more than 500 million daily active users [10]. A new anonymous account was created specifically for this study. Using the search term “kidney stone surgery”, the first 100 posts from individuals who were clearly identifiable as a patient were assessed. To be sure the subject was a patient, the individual had to state this explicitly in the content. Use of specific hashtags was not required. The search was conducted at a single time point for this group (July 1, 2023). A previous system developed by Ramkumar et al. [11] was applied as a framework for categorizing the information collected. Modifications were made to adjust for kidney stone surgery as the topic. Posts were evaluated for: (1) timing; (2) tone; (3) surgical procedure; (4) activities of daily life; (5) recovery; (6) return to work; (7) nervousness; (8) stent issues; (9) stent on a string; (10) diet and prevention; (11) pain; (12) multiple sessions; (13) gratitude for health care services; (14) disease recurrence; and (15) costs. Information was also collected on sex, geographical location, likes, and number of views (if video format).

The first 100 posts from self-identified urologists were also collected, along with the same data for patients. In these cases, the individual’s profile description had to state explicitly that they were a certified urologist in order to be eligible for inclusion. The following additional themes were also assessed: (1) self-promotion; (2) educational content; (3) technology; and (4) false information. For posts that were deemed to have educational intent, content was checked against the current European Association of Urology (EAU) guidelines and for inclusion of a study reference [12]. The search was conducted at a single time point for this group (July 2, 2023). Only one post was collected per user. All accounts viewed were public. Given that all the data are available freely in the public domain, ethical approval was not deemed necessary.

## 3 Results

### 3.1 Posts from patients

Some 71% of the patient group were female (Table 1). The majority of posts were from the USA (81%), followed by the UK (6%). The average numbers of likes and views were 76 (range 2–616) and 6421 (range 823–11 481), respectively. Regarding timing, 24% of the posts were preoperative, 24% were perioperative, and 52% were postoperative. The type of kidney stone surgery was not specified in most posts, but was mentioned as ureteroscopy (URS) in 22%, percutaneous nephrolithotomy (PCNL) in 10%, and shockwave lithotripsy in 2%.

**Table 1:** Summary of demographics and quality-of-life domains covered in Instagram posts

	Patients	Urologists
**Demographics**		
Mean number of views (range)	6421 (823–11 481)	3975 (81–37 100)
Mean number of likes (range)	76 (2–616)	324 (7–2869)
Sex (*n*)		
Male	29	94
Female	71	6
Top 3 countries of origin for post		
1.	USA	Turkey
2.	UK	USA
3.	India	Iran
**Quality-of-life domains covered**		
Activities of daily life	18%	0%
Recovery	42%	11%
Return to work	11%	0%
Nervousness	16%	0%
Stent issues	31%	3%
Stent on a string	5%	1%
Diet and prevention	9%	0%
Pain	43%	1%
Need for multiple sessions	45%	0%
Grateful	11%	1%
Recurrence	8%	1%
Cost	10%	0%

The tone of the majority of posts was negative (65%). The commonest themes covered in the patient posts were a need for multiple operative sessions (45%), pain (43%), and recovery (42%). The other themes included activities of daily life (18%), return to work (11%), nervousness (16%), stent issues (31%), stent on string (5%), diet and prevention (9%), gratitude for health care services (10%), disease recurrence (18%), and costs (10%). Some 57% of the posts covered more than one theme.

### 3.2 Posts from urologists

Some 94% of the posts by urologists were from males and the largest proportion (24%) came from Turkey.

**Fig. 1:**
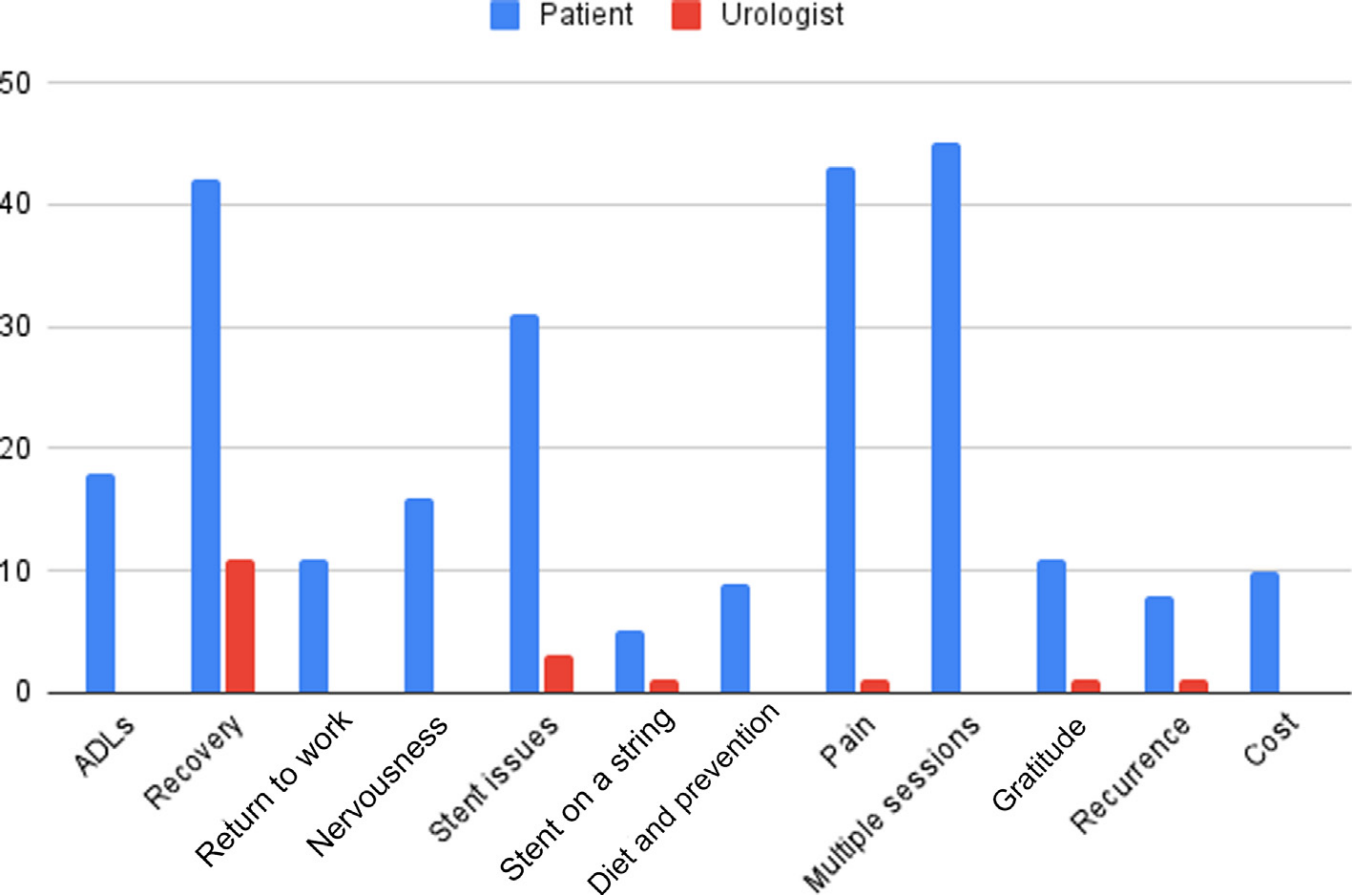
Comparison of quality-of-life domains covered in Instagram posts by patients and urologists. ADLs = activities of daily life.

The average numbers of likes and views were 324 (range 7–2869) and 3975 (range 81–37 100), respectively. Some 64% of the posts pertained to URS and 28% to PCNL. All of the posts from urologists were neutral in tone and 80% involved images or material taken during an operation. In comparison to the themes covered in the patient group, the distribution of content by domain was as follows: recovery, 11%; stent issues, 3%; pain, 1%; stent on a string, 1%; gratitude for health care services, 1%; and recurrence, 1% (Fig. 1). None of the posts addressed activities of daily life, costs, return to work, nervousness, diet and prevention, or the need for multiple operative sessions.

By contrast, the content of posts from urologists included self-promotion (79%) and new technology (52%). Overall, 17% of posts were educational. Some 10% of all the posts contained material that did not support current EAU guidelines and also contained false information. Examples include multiple posts stating that URS is completely free of complications and that PCNL can guarantee stone-free status. None of the posts included a study reference. Moreover, 83% of the posts contained surgical images. These were all anonymized except for three cases in which either the patient’s face (*n* = 2) or their name (*n* = 1) was shown. The majority of the posts with surgical images (97%) had no clear statement that patient consent had been obtained before posting.

## 4 Discussion

Our study reveals that many patients use social media as a platform for sharing their perspectives on kidney stone surgery. The commonest issues that patients highlight are the need for multiple operative sessions, pain related to surgery, and the recovery period. The majority of content posted by patients therefore relates to negative experiences. In stark contrast, posts from urologists seldom address the same issues. Instead, they focus on self-promotion and new technologies for lithotripsy. The commonest issue raised by patients was the need for multiple operative sessions, which was not addressed in any of the posts from urologists. Most of the latter included surgical images, but very few clearly stated that consent had been obtained. Furthermore, patients were identifiable in a minority of cases.

Over time there has been increase in focus on the impact of KSD on patient quality of life (QoL) and the impact of surgical treatments and ureteral stents [13], [14], [15]. There is also evidence of greater QoL impairment for younger patients and females [16]. Multiple patient-reported outcome measures have been developed to assess this issue and are increasingly being applied in clinical and research settings [17]. While the use of such tools in the clinical setting is valuable, there is also value in reviewing the unprompted feedback that patients share, such as on social media.

Given the number of new technologies being developed for lithotripsy, it is perhaps not surprising that this topic accounts for a large proportion of posts from urologists. Sharing of images and video footage of new surgical instruments would therefore seem to be a natural accompaniment. However, in the domain of social media, the practice patterns of health professionals lack the same regulations that are closely followed in scientific journals, such as the need for signed consent forms. Such has been the rapid expansion of social media that the professional challenges of upholding privacy, confidentiality, and content accuracy are yet to be met [18]. The EAU has produced best practice recommendations on appropriate use of social media, which include “thinking twice” or refraining from posting surgical images, keeping separate personal and professional profiles, and avoiding direct interactions with direct questions [19]. The data collection process for this study revealed many examples in which this guidance was not being followed by urologists. Similar research in other surgical disciplines confirms that this is not an issue limited to urology [20]. Most worrisome is arguably cases in which patient details have not been anonymized and/or the patient is identifiable. Regarding maintaining professional standards, the regulatory body that takes disciplinary action is usually the medical council in the country where the clinician is based. Kilic et al. [21] reported that in 2019, more than 1000 health care professionals were investigated for social media misconduct by General Medical Council in the UK. This resulted in at least two license suspensions and one permanent disqualification from clinical practice.

The discordance in content between patients and urologists reveals that there is much room for urologists to develop more tailored material for patient education. While it is generally accepted that social media can represent a valuable opportunity for patient education, examples of its successful use are currently lacking [18].

### 4.1 Limitations

Our study has several limitations that must be acknowledged. First, only public posts were reviewed, and it is possible that private posts would have provided more honest perspectives. In addition to this possible bias, there is a lack of research addressing why certain patients choose to post their views on surgery while others do not. However, given that social media can act as a vehicle for sharing many aspects of a person’s everyday life and particularly their emotions, it seems understandable that some may choose to share their experiences of surgery and that this practice will increase in the future. On other social media platforms such as Facebook where users can create formal groups, patients have set up support networks for particular medical conditions. There are reports that such groups (including open and private groups) are used as a means to reach such communities and to disseminate surveys [22]. This could be one way to overcome this bias and reach a larger network of patients.

We were not able to identify the age of individuals. Given that social networking sites are commonly associated with younger generations, it is possible that our results may be biased towards the perceptions of younger patients. The accuracy of the medical information was not assessed using a validated scale such as the DISCERN score; however, this was not the main focus of our study. We pooled all types of kidney stone surgery for the study, and responses were not broken down according to surgery type given the lack of information in patient posts. Over time, the search methods and selections on this platform have been subtly evolving, which could impact the reproducibility of our results.

## 5 Conclusions

Patients’ quality of life is affected across many areas when they undergo kidney stone surgery. Patients, particularly females, appear to use Instagram as an avenue to share their perspectives, mostly regarding negative experiences. By contrast, urologists, mostly males, mainly use Instagram as a means of self-promotion and to share their experiences with new technologies. This content does not match the themes raised by patients. It is highly concerning that there appears to be a low level of adherence to EAU recommendations regarding professional conduct on social media.

  ***Author contributions:*** Patrick Juliebø-Jones had full access to all the data in the study and takes responsibility for the integrity of the data and the accuracy of the data analysis.

  *Study concept and design*: Juliebø-Jones, Emiliani, Sierra, Esperto, Ventimiglia, Pietropaolo, Somani.

*Acquisition of data*: Juliebø-Jones, Sierra, Esperto, Ventimiglia, Pietropaolo, Tzelves.

*Analysis and interpretation of data*: Juliebø-Jones, Ventimiglia, Tzelves.

*Drafting of the manuscript*: Juliebø-Jones, Emiliani.

*Critical revision of the manuscript for important intellectual content*: Juliebø-Jones, Emiliani, Sierra, Esperto, Ventimiglia, Pietropaolo, Tzelves, Beisland, Somani.

*Statistical analysis*: None.

*Obtaining funding*: None.

*Administrative, technical, or material support*: Beisland.

*Supervision*: Beisland, Somani.

*Other*: None.

  ***Financial disclosures:*** Patrick Juliebø-Jones certifies that all conflicts of interest, including specific financial interests and relationships and affiliations relevant to the subject matter or materials discussed in the manuscript (eg, employment/affiliation, grants or funding, consultancies, honoraria, stock ownership or options, expert testimony, royalties, or patents filed, received, or pending), are the following: None.

  ***Funding/Support and role of the sponsor:*** None.

  ***Data sharing statement:*** The data sets generated and/or analyzed during the current study are available from the corresponding author on reasonable request.

  ***Ethical approval:*** Ethical approval was deemed not to be required given all the data are in the public domain and are anonymized.
